# Draft Genome Sequence of a Clinically Isolated Extensively Drug-Resistant *Pseudomonas aeruginosa* Strain

**DOI:** 10.1128/genomeA.00162-16

**Published:** 2016-03-24

**Authors:** Bhavani Manivannan, Niranjana Mahalingam, Sudhir Jadhao, Amrita Mishra, Pravin Nilawe, Bulagonda Eswarappa Pradeep

**Affiliations:** a Department of Biosciences, Sri Sathya Sai Institute of Higher Learning, Prasanthi Nilayam, India; b Department of Microbiology, Sri Sathya Sai Institute of Higher Medical Sciences, Prasanthigram, India; c Bionivid Technology Pvt. Ltd., Bengaluru, India; d Thermo Fisher Scientific, Mumbai, India

## Abstract

We present the draft genome assembly of an extensively drug-resistant (XDR) *Pseudomonas aeruginosa* strain isolated from a patient with a history of genito urinary tuberculosis. The draft genome is 7,022,546 bp with a G+C content of 65.48%. It carries 7 phage genomes, genes for quorum sensing, biofilm formation, virulence, and antibiotic resistance.

## GENOME ANNOUNCEMENT

*Pseudomonas aeruginosa* is an opportunistic human pathogen associated with several life-threatening infections including pneumonia, bacteremia, meningitis, cystic fibrosis, and urinary tract and wound infections. Although several genome sequences of *P. aeruginosa* are available in the GenBank database (1, 2), the enormous versatility of this pathogen to adapt and thrive in various ecological niches developing enhanced resistance to most of the available antibiotics necessitates sequencing of additional clinical isolates.

In this report, we present the draft genome sequence of an extensively drug-resistant (XDR) *P. aeruginosa* recovered from the urine sample of a 33-year old female patient with a history of genito urinary tuberculosis at Sri Sathya Sai Institute of Higher Medical Sciences, Prasanthigram, India. She had undergone a right ureteric reimplant for right lower ureteric stricture with proximal hydroureteronephrosis. This pathogen was found to be resistant to penicillins, second and third generation cephalosporins, aminoglycosides, carbapenems, tetracyclines, quinolones, and trimethoprim-sulfamethoxazole and sensitive to only colistin when tested with Vitek-2. Total DNA was extracted from an overnight culture of a single colony in Luria broth (LB) medium using a genomic DNA extraction kit (Macherey-Nagel, Germany).

Sequencing on Ion Torrent single-end technology produced a total of 1,022,829 bp single-end reads with an average read length of 271.50 ± 99.16 bp. Assembly by SPADES v3.5.0 (3) revealed that the draft genome consists of 190 scaffolds with an average length of 36,960.77 bp, G+C content of 65.68%, and *N*_50_ size of 65,43,920 bp constituting a total of 7,022,546 bp. Genome annotation by RAST (4) and NCBI PGAAP servers revealed that the draft genome has 7,803 protein coding sequences (CDSs), 3 rRNAs, 49 tRNAs, and 1 noncoding RNA (ncRNA) genes. No plasmid was identified when analyzed using Webcutter (v2.0) and Plasmid Finder (v1.3) (5). The contigs of the draft genome share (93.10%) identity with those of the *P. aeruginosa* 19BR genome having polymyxin-B adaptation (GenBank Accession no. AFXJ00000000) (6).

RAST annotation revealed the presence of several genes responsible for quorum sensing, biofilm formation, and virulence. Analysis by Resfinder 2.1 (7) indicates that the draft genome has genes encoding for resistance to aminoglycosides, fosfomycin, β-lactams, sulfonamides, and trimethoprim. Further, 7 putative prophage sequences were identified through the PHAST server (8). Taxonomy identification of the draft genome by EzTaxon (9) and MEGA6 inferred that *Pseudomonas aeruginosa* is the putative species (as per sequence homology) while exhibiting closest homology to *Pseudomonas otitidis*. A more detailed report of this strain will be included in a future publication.

### Nucleotide sequence accession numbers. 

The whole-genome shotgun project of this strain has been deposited at DDBJ/EMBL/GenBank under the accession no. LQBU00000000. The version described in this paper is LQBU00000000.1.
